# Microindolinone A, a Novel 4,5,6,7-Tetrahydroindole, from the Deep-Sea-Derived Actinomycete *Microbacterium* sp. MCCC 1A11207

**DOI:** 10.3390/md15070230

**Published:** 2017-07-19

**Authors:** Siwen Niu, Ting-Ting Zhou, Chun-Lan Xie, Gai-Yun Zhang, Xian-Wen Yang

**Affiliations:** 1 State Key Laboratory Breeding Base of Marine Genetic Resources, Key Laboratory of Marine Genetic Resources, Third Institute of Oceanography, State Oceanic Administration, 184 Daxue Road, Xiamen 361005, China; niusi123@126.com (S.N.); zhoutt@outlook.com (T.-T.Z.); xiechunlanxx@163.com (C.-L.X.); 2 Fujian Key Laboratory of Marine Genetic Resources, South China Sea Bio-Resource Exploitation and Utilization Collaborative Innovation Center, Third Institute of Oceanography, State Oceanic Administration, 184 Daxue Road, Xiamen 361005, China; zhgyun@tio.org.cn

**Keywords:** deep-sea, actinomycete, *Microbacterium* sp., indole

## Abstract

A novel indole, microindolinone A (**1**), was isolated from a deep-sea-derived actinomycete *Microbacterium* sp. MCCC 1A11207, together with 18 known compounds (**2**–**19**). By detailed analysis of the ^1^H, ^13^C, HSQC, COSY, HMBC, high resolution electron spray ionization mass spectrum (HRESIMS), and circular dichroism (CD) data, the absolute configuration of **1** was elucidated as 5*R*-hydroxy-4,5,6,7-tetrahydroindole-4-one. It is noteworthy that **1** is the second example of a saturated indole isolated from nature.

## 1. Introduction

Actinomycetes are Gram-positive bacteria known for their ability to produce structurally novel secondary metabolites with various biological activities [1]. The best-known compound is salinosporamide A [2,3]. Very recently, the representative examples included pyrazolofluostatins and aminorifamycins isolated from marine *Micromonospora* species [4,5].

The genus *Microbacterium* of the *Microbacteriaceae* family was first proposed by Orla-Jensen in 1919. Up to now, there are 97 species reported from diverse habitats including land, ocean, air, blood, etc. However, only four compounds were reported from this genus [6,7]. In our current research for novel compounds from deep-sea-derived microorganisms [8,9,10], the actinomycete *Microbacterium* sp. MCCC 1A11207, isolated from southwestern Indian Ocean sediment, was subjected to a systematic chemical examination. Consequently, one new and 18 known compounds were obtained. Herein, we report the isolation, structural elucidation, and bioactivities of these compounds.

## 2. Results and Discussion

**Figure 1:**
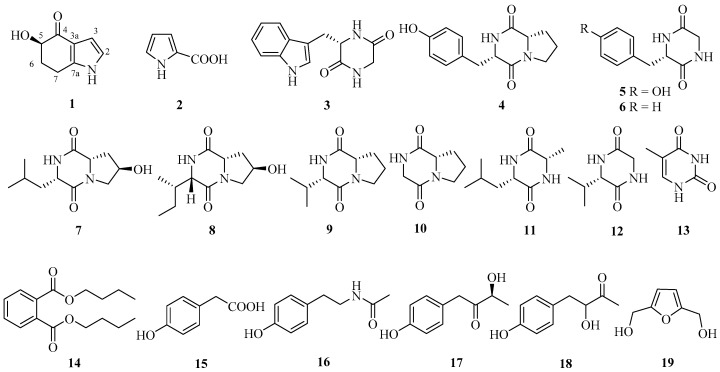
Compounds isolated from *Microbacterium* sp. MCCC 1A11207.

*Microbacterium* sp. MCCC 1A11207 was cultured in a 50 L fermentor containing 30 L A3 medium for 10 days. Then, the fermentation broth was centrifuged. The supernatant was extracted with EtOAc and the mycelium was extracted with MeOH. They were concentrated and combined to give the crude extract (17 g). By repeated column chromatography (CC) over silica gel, octadecylsilyl (ODS), and Sephadex LH-20, 19 compounds were obtained (Figure 1).

### 2.1. Structure Elucidation

**Figure 2:**
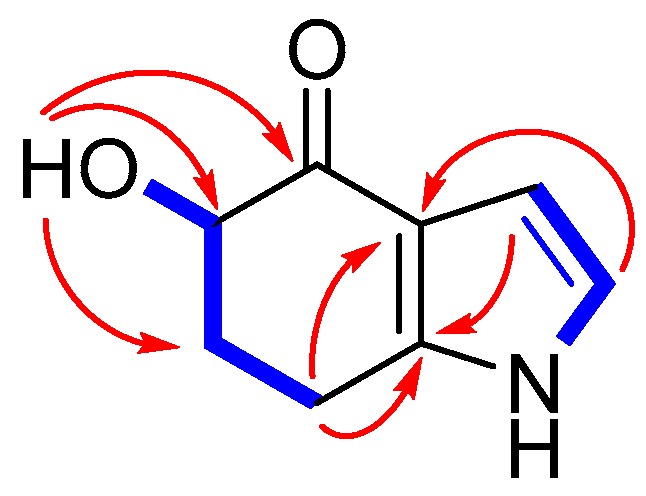
Key ^1^H–^1^H COSY (bold) and HMBC (arrow) correlations of **1**.

Microindolinone A (**1**) was isolated as a colorless oil. The molecular formula was established as C_8_H_9_NO_2_ on the basis of a sodium adduct ion peak at *m*/*z* 174.0525 [M + Na]^+^ in its HRESIMS, requiring five degrees of unsaturation. The ^1^H-NMR spectrum (Figure S1) exhibited two exchangeable protons at *δ*_H_ 11.3 (1H, brs, 1-NH) and 4.98 (1H, d, *J* = 3.8 Hz, 5-OH), one oxygenated *sp*^3^ (*δ*_H_ 4.05, ddd, *J* = 11.6, 4.5, 3.8 Hz, H-5) and two *sp*^2^ [(*δ*_H_ 6.74, dd, *J* = 2.9, 2.4 Hz, H-2) and (*δ*_H_ 6.25, dd, *J* = 2.9, 2.2 Hz, H-3)] methines, together with two methylenes. The ^13^C attached proton test (APT)-NMR spectrum (Figure S2) showed eight resonance signals involving three non-protonated carbons at *δ*_C_ 194.1 (C-4), 143.4 (C-7a), and 118.4 (C-3a), three methines (*δ*_C_ 120.3/C-2, 105.2/C-3, and 72.6/C-5), and two methylenes at *δ*_C_ 33.0 (C-6) and 21.3 (C-7) (Table 1). In the ^1^H–^1^H COSY spectrum (Figure S4), two fragments of NH-1/C-2/C-3 and OH-5/C-5/C-6/C-7 were determined on the basis of COSY correlations of NH-1(*δ*_H_ 11.3)/H-2 (*δ*_H_ 6.74)/H-3 (*δ*_H_ 6.25), and 5-OH (*δ*_H_ 4.98)/H-5 (*δ*_H_ 4.05)/H_2_-6 (*δ*_H_ 1.87, m; 2.20, m)/H_2_-7 (*δ*_H_ 2.83, m) (Figure 2). These two fragments can be connected by a α,β-unsaturation ketone unit on the basis of HMBC cross-peaks (Figure S5) from H-2 to C-3/C-3a, H-3 to C-7a, OH-5 to C-4/C-5/C-6, and H_2_-7 to C-3a/C-5/C-6/C-7a (Figure 2), which established the planar structure of **1** as 5-hydroxy-4,5,6,7-tetrahydroindole-4-one.

**Figure 3:**
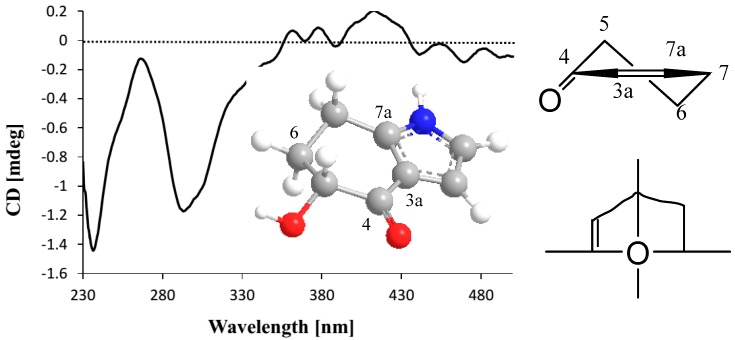
The CD spectrum in CHCl_3_ and octant projection of compound **1**.

The large coupling constant of H-5 and H-6a (*J*_H-5/H-6a_ = 11.6 Hz) indicated H-5 as axial-orientation. In the CD spectrum, the negative Cotton effect (Δε_29__6_ −0.35) induced by n-π* electronic transition revealed the *R*-orientation of the 5-hydroxyl group on the basis of the octant rule (Figure 3) [11,12]. Therefore, the absolute configuration of **1** was determined as 5*R*-hydroxy-4,5,6,7-tetrahydroindole-4-one, and named microindolinone A. Surprisingly, although indoles occur broadly in nature [13,14,15], the saturated ones were seldom discovered. As a matter of fact, the only one reported was 6,7-dihydroxy-4,5,6,7-tetrahydroindole-4-one from *Nocardia* sp. (a soil-derived actinomycete) [16], and microindolinone A (**1**) was the second example. It is noteworthy that the absolute configuration of this saturated indole was determined for the first time.

By comparing the ^1^H-, ^13^C-NMR, MS, and optical rotation (OR) data with those reported in the literature, 18 known compounds were identified as pyrrole-2-carboxylic acid (**2**) [17], cyclo(l-Trp-Gly) (**3**) [18], cyclo-l-Tyr-l-Pro (**4**) [19], cyclo(l-Trp-Gly) (**5**) [20], cyclo(l-Phe-Gly) (**6**) [21], cyclo[l-(4-hydroxyprolinyl)-l-leucine] (**7**) [22,23], cyclo[l-(4-hydroxyprolinyl)-l-isoleucine] (**8**) [24], cyclo-(l-Pro-l-Val) (**9**) [25], cyclo-(l-Pro-Gly) (**10**) [25], cyclo-(l-Leu-l-Ala) (**11**) [26], cyclo-(l-Val-Gly) (**12**) [27], 5-methyluracil (**13**) [26], dibutyl phthalate (**14**) [28], 4-hydroxyphenylacetic acid (**15**) [29], *N*-(4-hydroxyphenyl)-acetamide (**16**) [30], (*S*)-3-hydroxy-4-(4-hydroxyphenyl)butan-2-one (**17**) [31], 3-hydroxy-4-(4-dihydroxyphenyl)-2-butanone (**18**) [32], and (5-hydroxymethyl-furan-2-yl)-methanol (**19**) [33]. Surprisingly, these 19 compounds were all firstly isolated from *Microbacterium* species.

### 2.2. Anti-Proliferative Activity of ***1*** against RBL-2H3 Cells

Microindolinone A (**1**) was tested for anti-proliferative activity against RBL-2H3 cells. Fortunately, it did not show significant cytotoxicity, even under the highest concentration of 20 µg/mL (Table 2).

### 2.3. Anti-Allergic Activity of ***1***

Microindolinone A (**1**) was further subjected to anti-allergic bioactivity on IgE-mediated rat mast RBL-2H3 cells. However, it did not show any positive effects under the concentration of 20 μg/mL, while the positive control loratadine exhibited a significant inhibition rate of 37% (Table 3).

## 3. Materials and Methods

### 3.1. General Experimental Procedures

HRESIMS spectra were obtained from a Xevo G2 Q-TOF mass spectrometer (Waters). Optical rotations were conducted by a Rudolph IV Autopol automatic polarimeter. NMR spectra were recorded on a Bruker 400 MHz spectrometer. Materials for column chromatography were silica gel (Qingdao Marine Chemistry Co. Ltd., Qingdao, China), ODS (50 μm, Daiso, Japan), and Sephadex LH-20 (Amersham Pharmacia Biotech AB, Uppsala, Sweden). Pre-coated silica gel plates were used for thin-layer chromatography (TLC) analysis.

### 3.2. Bacterial Material

The strain MCCC 1A11207 was isolated from a deep-sea sediment of the southwestern Indian Ocean (−1603 m) in 2014. By comparison of its 16S rRNA gene sequence with those of validly published names from the GenBank database via the BLAST program, the strain showed the highest similarity (98.03%) to *Microbacterium amylolyticum* N5^T^. Therefore, it was identified to be *Microbacterium* sp. MCCC 1A11207. A voucher strain of the actinomycete was deposited in the Marine Culture Collection of China with the accession number of MCCC 1A11207.

### 3.3. Cultivation and Extraction

The strain was cultured on the 2216E medium at 28 °C for 3 days and the colony was inoculated to 250 mL Erlenmeyer flasks containing 50 mL A3 medium compositing with 15 g bacterial peptone, 5 g soybean peptone, 15 g soluble starch, 30 g marine salt, and 1 L tap water, and then was cultured in a rotary shaker with 180 rpm at 28 °C for 3 days as the spores’ medium. The large-scale fermentation was performed by a 50 L fermentor containing 30 L A3 medium with the 5% seed culture, and the fermentation continued at 28 °C with 180 rpm for 10 days. Then, the fermentation broth was centrifuged (16,000 rpm) to get supernatant and mycelium. The supernatant was extracted with EtOAc three times, and then concentrated under reduced pressure to provide the crude extract A. The mycelium was extracted with MeOH twice. After removing MeOH, the residue was re-extracted with EtOAc three times to get extract B under reduced vacuum. Extracts A and B were combined to give the total crude extract.

### 3.4. Isolation and Purification

The total crude extract (17 g) was subjected to column chromatography (CC) on ODS, eluting with a gradient MeOH–H_2_O (5:95→100:0) to give four fractions (Fr.1–Fr.4). Fraction Fr.2 (92 mg) was first subjected to Sephadex LH-20 CC eluting with MeOH, and then by CC over silica gel using CHCl_3_–MeOH (100:1) to provide **2** (6.8 mg). Fraction Fr.3 (283 mg) was separated by CC over Sephadex LH-20 (MeOH) to get five subfractions (Fr.3.1–Fr.3.5). Subfraction Fr.3.1 was purified by CC on silica gel eluting with petroleum ether (PE)–acetone (2:1) to get **14** (11.3 mg). Compounds **12** (9.2 mg) and **19** (2.3 mg) were isolated from subfraction Fr.3.2 by CC over silica gel (CHCl_3_–MeOH, 20:1), while **5** (2.1 mg) and **13** (23.0 mg) were obtained from subfraction Fr.3.5 (CHCl_3_–MeOH, 6:1). Compound **10** (38.0 mg) was isolated from Fr.3.3 using recrystallization in MeOH. Subfraction Fr.3.4 was subjected to CC over silica gel eluting with PE–acetone (3:1) to get **1** (1.1 mg). Fraction Fr.4 (380 mg) was fractionated by CC on Sephadex LH-20 (MeOH) to obtain five subfractions (Fr.4.1–Fr.4.5). Subfraction Fr.4.1 was subjected to CC over silica gel (PE–acetone, 2:1) to get two subfractions Fr.4.1.1 and Fr.4.1.2. Subfraction Fr.4.1.1 was purified by CC over silica gel (PE–acetone, 2:1) to get **11** (1.8 mg). Fr.4.1.2 was subjected to MPLC using gradient MeOH–H_2_O (5→30%) to get **7** (8.0 mg), **8** (3.8 mg), and **9** (8.7 mg). Fr.4.2 was purified by CC over silica gel (CHCl_3_–MeOH, 6:1) to provide **16** (38.2 mg). Fr.4.3 was subjected to CC on silica gel (CHCl_3_–MeOH, 20:1) to get two subfractions Fr.4.3.1 and Fr.4.3.2. Subfraction Fr.4.3.1 was further purified by Prep. TLC (PE–EtOAc, 1:1) to get **17** (1.2 mg) and **18** (1.4 mg), while compounds **4** (12.1 mg) and **6** (9.8 mg) were isolated from Fr.4.3.2 by CC over silica gel (PE–EtOAc, 1:1). Fr.4.4 and Fr.4.5 were purified by CC on silica gel eluting with PE–EtOAc (1:1) and PE–EtOAc (2:1) to get **15** (8.6 mg) and **3** (2.3 mg), respectively.

*Microindolinone A* (**1**): Colorless oil; ${\left[\alpha \right]}_{D}^{25}$ +2.5 (*c* 0.11, MeOH); CD (CHCl_3_) λ_max_ (Δε) 237 (−0.42), 268 (−0.04), 296 (−0.35); ^1^H- and ^13^C-NMR data, see Table 1; HRESIMS (positive) *m*/*z* 174.0525, calcd. for C_8_H_9_NO_3_Na^+^ 174.0531.

### 3.5. Anti-Proliferative Assay

According to previously reported protocols [34], the cytotoxicity test was carried out using the MTT assay on RBL-2H3 cells. In brief, RBL-2H3 cells were seeded into 96-well cell culture plates. Then, six different concentrations of **1**, ranging from 0.625 to 20 µg/mL, were added. After 24 h, the cells were treated with 20 µL MTT solution. The cytotoxicity was quantified by measuring the absorbance at 570 nm. The cell viability was calculated using the following equation: Cell viability (%) = [(As − Av)/(Ac − Av) × 100%, where As is the absorbance of the sample, Av is the absorbance of the vehicle, and Ac is the absorbance of the control.

### 3.6. Anti-Allergic Test

The anti-allergic activity, indexed by the β-hexosaminidase release, was measured for the efficiency of the RBL-2H3 cell degranulation inhibition rate using IgE-mediated mast cell allergic reaction [8,35]. In short, RBL-2H3 cells were seeded into 96-well cell culture plates (1 × 10^5^ cells/well) to incubate with dinitrophenol (DNP) specific IgE overnight. IgE-sensitized RBL-2H3 cells were pre-treated with compound **1** (20 μg/mL) for 1 h and stimulated with dinitrophenyl-bovine serum albumin (DNP-BSA) (500 ng/mL). The negative control group was added to 200 μL phosphate-buffered saline (PBS) buffer. The β-hexosaminidase activity was quantified by measuring the fluorescence intensity of the hydrolyzed substrate in a fluorometer. The degranulation efficiency was calculated using the following formula: Degranulation efficiency (%) = Fsup/(Fsup + Flys) × 100%, where Fsup is the fluorescence value of the supernatant and Flys is the fluorescence value of cell lysates. The inhibition rate was calculated based on the following formula: Inhibition rate (%) = (Positive − Sample)/(Positive − Negative) × 100%, where Positive is the degranulation efficiency of the DNP-BSA stimulated group, Sample is the degranulation efficiency of the sample group, and Negative is the degranulation efficiency of the vehicle group.

### 3.7. Statistical Analysis

Anti-proliferative and anti-allergic experiments were conducted three times. Results are presented as means ± SD. One-way analysis of variance (one-way ANOVA) comparison tests of SPSS was used to evaluate the statistical significances of the differences between experimental groups. Differences were considered statistically significant for *p* < 0.05 using Duncan’s multiple range tests between groups.

## 4. Conclusions

From the deep-sea-derived rare actinomycete *Microbacterium* sp. MCCC 1A11207, 19 secondary metabolites were isolated and identified. The new compound, microindolinone A (**1**), was determined as 5*R*-hydroxy-4,5,6,7-tetrahydroindole-4-one. It was the second example of the tetrahydroindole found in nature. Its absolute configuration was determined for the first time.

## Figures and Tables

**Table 1:** The ^1^H (400 MHz) and ^13^C (100 MHz) NMR spectroscopic data for **1** in DMSO-*d*_6_.

Position	*δ*_C_, Type	*δ*_H_ (*J* in Hz)
1		11.3, brs
2	120.3, CH	6.74, dd (2.9, 2.4)
3	105.2, CH	6.25, dd (2.9, 2.2)
3a	118.4, C	
4	194.1, C	
5	72.6, CH	4.05, ddd (11.6, 4.5, 3.8)
6	33.0, CH_2_	1.87, m; 2.20, m
7	21.3, CH_3_	2.83, m
7a	143.4, C	
5-OH		4.98, d (3.8)

**Table 2:** Anti-proliferative activity of **1** against RBL-2H3 cells (*n* = 3, means ± SD).

Concentrations (µg/mL)	Cell Viability (%)
20	91 ± 10
10	93 ± 1.4
5	90 ± 10
2.5	93 ± 12
1.25	94 ± 12
0.625	99 ± 14

**Table 3:** The Anti-allergic activity of **1** against RBL-2H3 cells (*n* = 3, means ± SD).

Compound	Concentration (μg/mL)	Inhibition Rate (%)
**1**	20	−1.4 ± 0.8
Loratadine	20	37 ± 5.3

## Supplementary Materials

The following documents are available online at www.mdpi.com/1660-3397/15/7/230/s1.
