# Supplementary material for: Microindolinone A, a Novel 4,5,6,7-Tetrahydroindole, from the Deep-Sea-Derived Actinomycete Microbacterium sp. MCCC 1A11207
Source: Mar Drugs. 2017 Jul 19;15(7):230. doi: 10.3390/md15070230 (PMC5532672; doi:10.3390/md15070230)
Supplement: Supplementary file 1 [file marinedrugs-15-00230-s001.pdf]

4-2

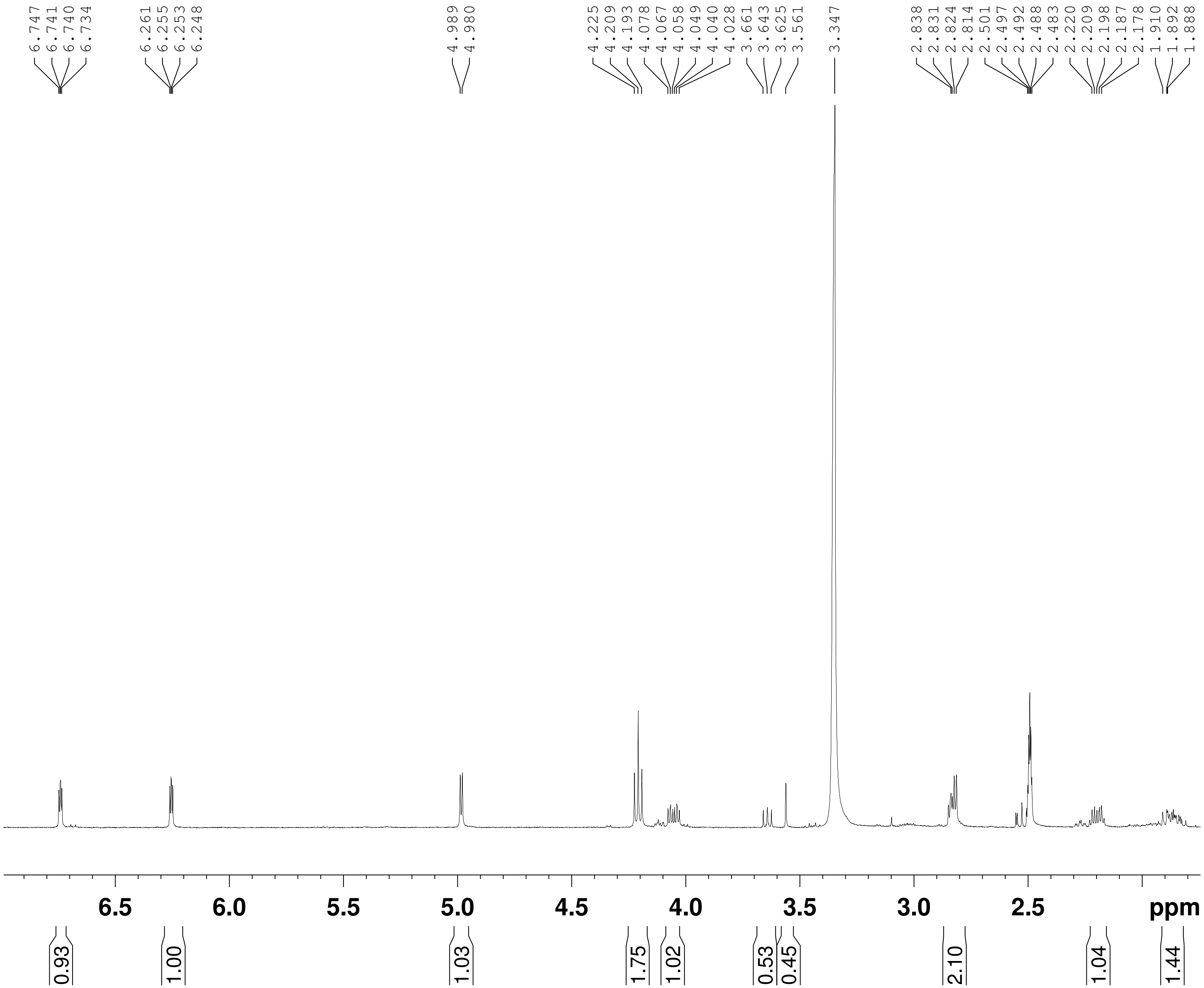


```
Current Data Parameters
NAME              TW43-4-6
EXPNO                    1
PROCNO                   1

F2 - Acquisition Parameters
Date_             20151106
Time                 23.55
INSTRUM              spect
PROBHD    5 mm PABBO BB-
PULPROG               zg30
TD                   65536
SOLVENT               DMSO
NS                      16
DS                       2
SWH               8223.685 Hz
FIDRES            0.125483 Hz
AQ               3.9845889 sec
RG                    50.8
DW                  60.800 usec
DE                    6.50 usec
TE                   293.9 K
D1              1.00000000 sec
TD0                      1

======== CHANNEL f1 ========
NUC1                    1H
P1                   13.09 usec
PL1                  -1.00 dB
PL1W           12.14314651 W
SFO1           400.1324710 MHz

F2 - Processing parameters
SI                   32768
SF             400.1300000 MHz
WDW                     EM
SSB      0
LB                    0.30 Hz
GB       0
PC                    1.00
```

4-2

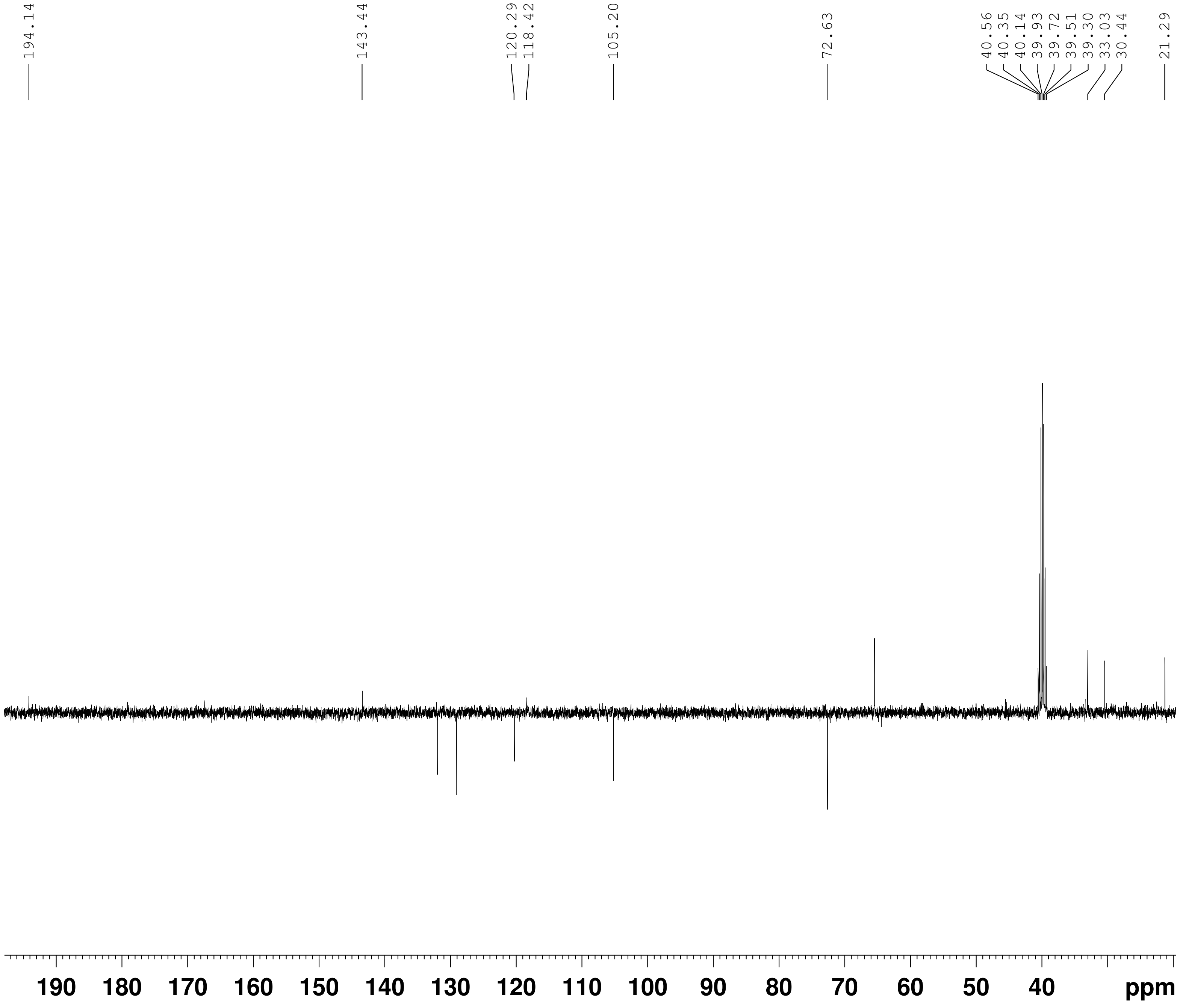

BRUKER

Current Data Parameters

NAME	TW43-4-6
EXPNO	2
PROCNO	1

F2 - Acquisition Parameters

Date_	20151107
Time	0.21
INSTRUM	spect
PROBHD	5 mm PABBO BB-
PULPROG	jmod
TD	65536
SOLVENT	DMSO
NS	3072
DS	4
SWH	24038.461 Hz
FIDRES	0.366798 Hz
AQ	1.3631488 sec
RG	203
DW	20.800 usec
DE	6.50 usec
TE	294.9 K
CNST2	145.0000000
CNST11	1.0000000
D1	2.00000000 sec
D20	0.00689655 sec
TD0	1

======== CHANNEL f1 ========

NUC1	13C
P1	12.37 usec
P2	24.74 usec
PL1	1.00 dB
PL1W	28.13319778 W
SFO1	100.6228298 MHz

======== CHANNEL f2 ========

CPDPRG[2	waltz16
NUC2	1H
PCPD2	80.00 usec
PL2	-1.00 dB
PL12	14.72 dB
PL2W	12.14314651 W
PL12W	0.32533529 W
SFO2	400.1316005 MHz

F2 - Processing parameters

SI	32768
SF	100.6127690 MHz
WDW	EM
SSB	0
LB	1.00 Hz
GB	0
PC	1.40

4-2

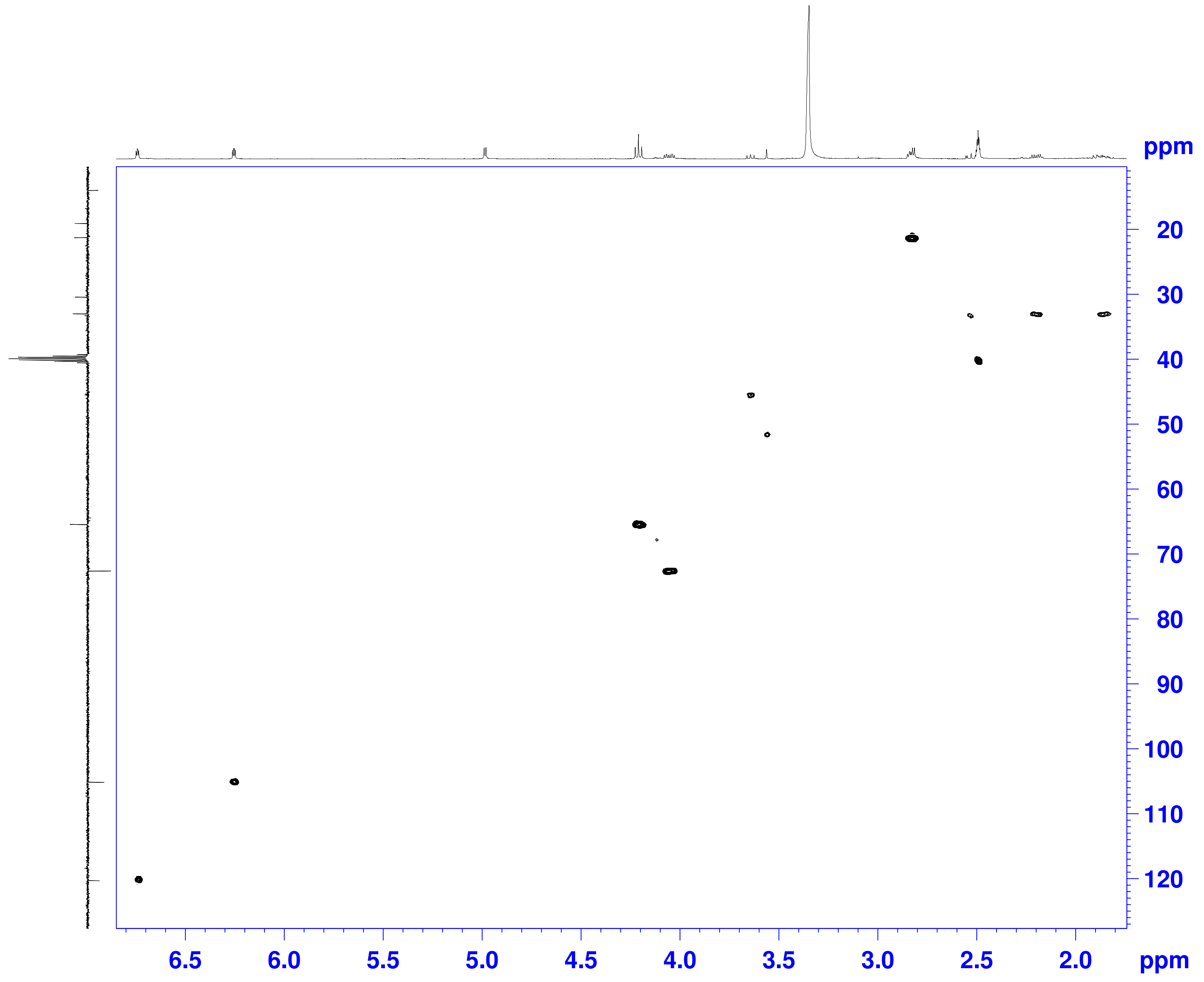


```
Current Data Parameters
NAME             TW43-4-6
EXPNO                   3
PROCNO                  1

F2 - Acquisition Parameters
Date_            20151107
Time                 2.55
INSTRUM             spect
PROBHD   5 mm PABBO BB-
PULPROG       hsqcedetgp
TD                   1024
SOLVENT              DMSO
NS                      8
DS                     16
SWH              4000.000 Hz
FIDRES           3.906250 Hz
AQ              0.1280000 sec
RG                    203
DW                125.000 usec
DE                   6.50 usec
TE                  294.6 K
CNST2         145.0000000
D0             0.00000300 sec
D1             1.50000000 sec
D4             0.00172414 sec
D11            0.03000000 sec
D13            0.00000400 sec
D16            0.00020000 sec
D21            0.00345000 sec
IN0            0.00003105 sec
ZGOPTNS

======== CHANNEL f1 ========
NUC1                   1H
P1                  13.09 usec
P2                  26.18 usec
P28                  1.00 usec
PL1                 -1.00 dB
PL1W          12.14314651 W
SFO1          400.1320007 MHz

======== CHANNEL f2 ========
CPDPRG[2             garp
NUC2                  13C
P3                  12.37 usec
P4                  24.74 usec
PCPD2               75.00 usec
PL2                  1.00 dB
PL12                16.65 dB
PL2W          28.13319778 W
PL12W          0.76598305 W
SFO2          100.6208180 MHz

====== GRADIENT CHANNEL =====
GPNAM[1]         SINE.100
GPNAM[2]         SINE.100
GPZ1                80.00 %
GPZ2                20.10 %
P16               1000.00 usec

F1 - Acquisition parameters
TD                    256
SFO1             100.6208 MHz
FIDRES          62.888012 Hz
SW                160.000 ppm
FnMODE       Echo-Antiecho

F2 - Processing parameters
SI                   1024
SF            400.1300000 MHz
WDW                 QSINE
SSB                     2
LB             0 Hz
GB             0
PC                   1.40

F1 - Processing parameters
SI                   1024
MC2         echo-antiecho
SF            100.6127690 MHz
WDW                 QSINE
SSB                     2
LB             0 Hz
GB             0
```

4-2

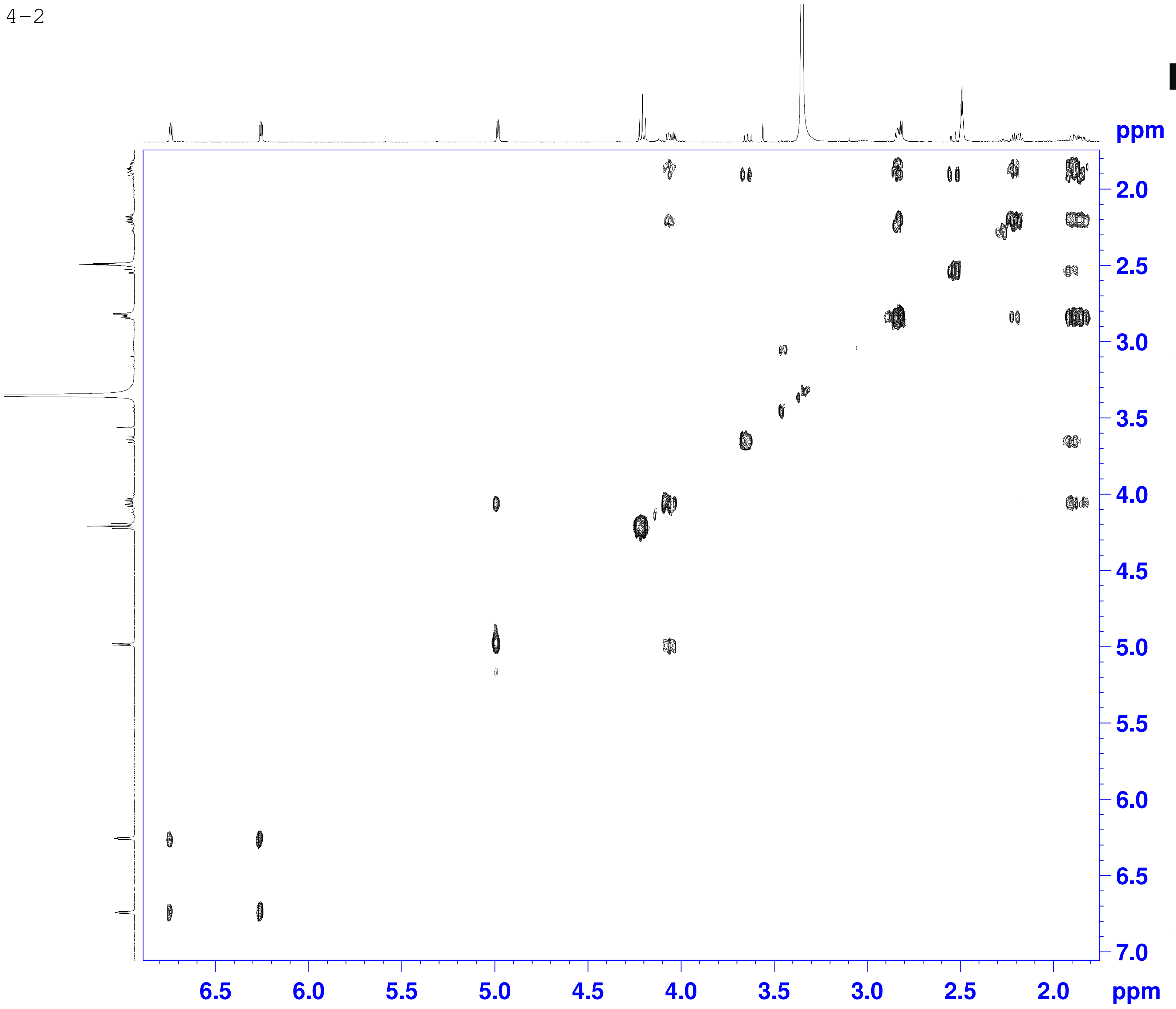

Current Data Parameters

NAME	TW43-4-6
EXPNO	4
PROCNO	1

F2 - Acquisition Parameters

Date_	20151107
Time	3.52
INSTRUM	spect
PROBHD	5 mm PABBO BB-
PULPROG	cosygpmfqf
TD	2048
SOLVENT	DMSO
NS	6
DS	8
SWH	4000.000 Hz
FIDRES	1.953125 Hz
AQ	0.2560000 sec
RG	203
DW	125.000 usec
DE	6.50 usec
TE	294.0 K
D0	0.00000300 sec
D1	1.93569195 sec
D13	0.00000400 sec
D16	0.00020000 sec
IN0	0.00024990 sec

======== CHANNEL f1 ========

NUC1	1H
P1	13.09 usec
PL1	-1.00 dB
PL1W	12.14314651 W
SFO1	400.1320007 MHz

====== GRADIENT CHANNEL =====

GPNAM[1]	SINE.100
GPNAM[2]	SINE.100
GPNAM[3]	SINE.100
GPZ1	16.00 %
GPZ2	12.00 %
GPZ3	40.00 %
P16	1000.00 usec

F1 - Acquisition parameters

TD	128
SFO1	400.132 MHz
FIDRES	31.260313 Hz
SW	10.000 ppm
FnMODE	QF

F2 - Processing parameters

SI	1024
SF	400.1299958 MHz
WDW	SINE
SSB	0
LB	0 Hz
GB	0
PC	1.40

F1 - Processing parameters

SI	1024
MC2	QF
SF	400.1299958 MHz
WDW	SINE
SSB	0
LB	0 Hz
GB	0

4-2

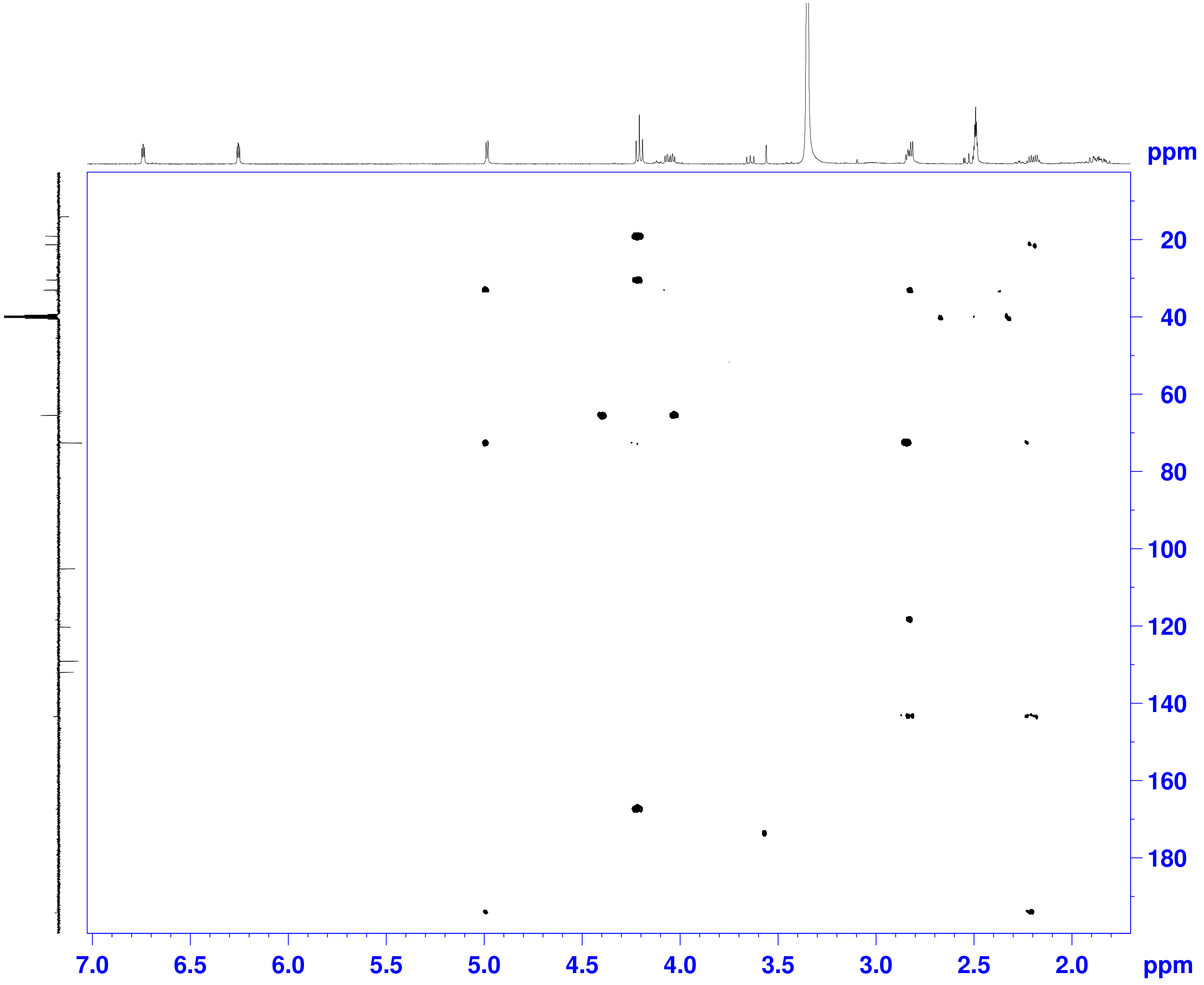

Current Data Parameters
NAME TW43-4-6
EXPNO 5
PROCNO 1

F2 - Acquisition Parameters
Date_ 20151107
Time 4.22
INSTRUM spect
PROBHD 5 mm PABBO BB-
PULPROG hmbcgpndqf
TD 4096
SOLVENT DMSO
NS 56
DS 16
SWH 4000.000 Hz
FIDRES 0.976563 Hz
AQ 0.5120000 sec
RG 203
DW 125.000 usec
DE 6.50 usec
TE 293.9 K
CNST13 8.0000000
D0 0.00000300 sec
D1 1.37220395 sec
D6 0.06250000 sec
D16 0.00020000 sec
IN0 0.00002485 sec

======== CHANNEL f1 ========
NUC1 1H
P1 13.09 usec
P2 26.18 usec
PL1 -1.00 dB
PL1W 12.14314651 W
SFO1 400.1320007 MHz

======== CHANNEL f2 ========
NUC2 13C
P3 12.37 usec
PL2 1.00 dB
PL2W 28.13319778 W
SFO2 100.6228303 MHz

====== GRADIENT CHANNEL =====
GPNAM[1] SINE.100
GPNAM[2] SINE.100
GPNAM[3] SINE.100
GPZ1 50.00 %
GPZ2 30.00 %
GPZ3 40.10 %
P16 1000.00 usec

F1 - Acquisition parameters
TD 128
SFO1 100.6228 MHz
FIDRES 157.223175 Hz
SW 200.000 ppm
FnMODE QF

F2 - Processing parameters
SI 1024
SF 400.1299958 MHz
WDW SINE
SSB 0
LB 0 Hz
GB 0
PC 1.40

F1 - Processing parameters
SI 1024
MC2 QF
SF 100.6127690 MHz
WDW SINE
SSB 0
LB 0 Hz
GB 0
